# Prevalence of hypertension in the Gambia and Sierra Leone, western Africa: a cross-sectional study

**DOI:** 10.5830/CVJA-2014-058

**Published:** 2014

**Authors:** Morcos Awad, Saman Setareh-Shenas, J Robert Pixton, Camelia Soliman, Lawrence SC Czer, Andrea Ruzza, James Mirocha

**Affiliations:** Division of Cardiology, Cedars-Sinai Heart Institute, Los Angeles, California; Division of Cardiology, Cedars-Sinai Heart Institute, Los Angeles, California; Division of Cardiology, Cedars-Sinai Heart Institute, Los Angeles, California; Division of Cardiology, Cedars-Sinai Heart Institute, Los Angeles, California; Division of Cardiology, Cedars-Sinai Heart Institute, Los Angeles, California; Division of Cardiothoracic Surgery, Cedars-Sinai Heart Institute, Los Angeles, California; Section of Biostatistics, Cedars-Sinai Medical Center, Los Angeles, California

**Keywords:** hypertension, the Gambia, Sierra Leone, prevalence, sodium, age, gender

## Abstract

**Background:**

Hypertension (HTN) is one of the causes of cardiovascular disease (CVD) in Africa, and may be associated with lower socio-economic status (SES). The prevalence of HTN is not well established in the Gambia or in Sierra Leone.

**Methods:**

A cross-sectional, population-based study of adults was conducted in the Gambia in 2000 and in Sierra Leone from 2001 to 2003 and in 2009. The study was conducted as part of the annual visit to countries in western Africa sponsored by a medical delegation from California. People from the Gambia and Sierra Leone were examined by the medical delegation and blood pressures were measured.

**Results:**

A total of 2 615 adults were examined: 1 400 females and 1 215 males. The mean systolic blood pressure (SBP) of the females was 134.3 ± 29.7 mmHg, mean diastolic blood pressure (DBP) was 84.5 ± 17.5 mmHg, and 46.2% were hypertensive. The mean SBP of the males was 132.8 ± 28.5 mmHg, mean DBP was 82.8 ± 16.2 mmHg, and 43.2% were hypertensive. Overall prevalence of HTN in the subjects was 44.8%. Mean SBP, mean DBP and HTN prevalence increased with age decade, both in males and females. In addition, after age adjustment (known age), females had higher mean SBP (*p* = 0.042), mean DBP (*p* = 0.001) and rate of occurrence of HTN (*p* = 0.016) when compared with males.

**Conclusions:**

Prevalence rates of HTN in the Gambia and Sierra Leone were higher than 40% in males and females, and may be a major contributor to CVD in both countries. Due to the association of HTN with low SES, improvements in educational, public health, economic, non-governmental and governmental efforts in the Gambia and Sierra Leone may lead to a lower prevalence of HTN. The cause of the higher prevalence in women may be due to post-menopausal hormonal changes.

## Abstract

Hypertension (HTN) is a chronic, slowly progressive disease affecting about one billion people globally and leading to about 7.1 million deaths annually. People of African origin may be particularly susceptible to hypertension.^1^-^3^ Defined as a sustained systolic blood pressure (SBP) above 140 mmHg, a diastolic blood pressure (DBP) above 90 mmHg or both, the aetiology of HTN can be classified as primary or secondary. While there is no known cause for primary (essential) HTN, which accounts for 90–95% of cases, the remaining 5–10% of cases is defined as secondary HTN and is caused by other disease conditions, which may affect the renal, circulatory, endocrine or other organ systems.

Many factors are associated with, and may contribute to the development and persistence of primary HTN, including obesity, stress, smoking,^4^ low potassium intake, high sodium (salt) and alcohol intake,^5^,^6^ familial and genetic influences,^7^,^8^ and low birth weight.^9^ On the other hand, hyperthyroidism, hypothyroidism and other conditions causing hormonal changes may be associated with primary pulmonary HTN.^10^,^11^ Regardless of the cause, the consequences of HTN include renal failure, heart failure, myocardial infarction, pulmonary oedema and stroke.^12^

Given these undesirable outcomes, treatment and prevention have assumed increasing emphasis in the management of HTN. Modification of risk factors can be achieved by reducing body weight and decreasing sugar intake, along with lowering alcohol consumption,^13^,^14^ as well as reducing salt intake and increasing potassium intake.^15^,^16^ Secondary HTN is managed by treating the underlying cause. Drugs available for the treatment of HTN, whether primary or secondary, include calcium-channel blockers (CCB), angiotensin converting enzyme inhibitors (ACEI), angiotensin receptor blockers (ARB), diuretics, α-blockers and β-blockers.

Race and ethnicity may influence pathogenesis, prevalence and treatment of HTN,^17^ perhaps through genetic influences. As a consequence, HTN remains one of the most common CVDs in Africa and one of the most frequent causes of death in the sub-Saharan African region.^18^,^19^ In 2000, the rate of HTN in sub-Saharan Africa was reported to be 26.9% in males and 28.3% in females.^20^ Low socio-economic status (SES) may additionally play an important role in the high prevalence of HTN in western and sub-Saharan Africa.

A cross-sectional survey in Tanzania revealed that treatment rates for HTN were very low, especially among people with low SES.^21^ Low SES led to inadequate education levels as a factor correlating with a higher blood pressure (BP) in adults and resulted in a low treatment rate for HTN due to monetary issues.^22^

Stress, in addition, was another factor related to HTN prevalence, especially in Africa.^23^ It has been shown that psychosocial stress affects the L-arginine/nitric oxide (NO) system, with a higher susceptibility in black Africans, which in turn contributes to a higher risk of CVD in those individuals.^24^

Therefore, a multiplicity of factors may be associated with and contributing to a high prevalence of HTN among Africans. The current study was undertaken to determine and quantitate the prevalence of HTN in two countries in western sub-Saharan Africa, namely, the Gambia and Sierra Leone.

## Methods

This was a population-based, cross-sectional study performed in the Gambia and Sierra Leone. The data were collected from the Gambia in 2000 and from Sierra Leone from 2001 to 2003 and in 2009. The Gambia is a small country, about 11 000 km^2^ in 2007, with a population of 1 705 000 by 2009.^25^,^26^ Sierra Leone is a larger country, about 72 000 km2 in 2007, with a population of 5 696 000 by 2009.^25^,^26^

This study took place as part of the annual visit to countries in western Africa sponsored by a medical delegation from California. In the Gambia, the visit was to specific areas within the capital city of Banjul, including Serrekunda, Latrikunda and Fajikunda. In Sierra Leone, the medical delegation visited Freetown, Kenema, Lunsar, Bonthe, Bo, Jui and Makeni.

People waited in queues to be examined in a clinic by the team.^27^ Subjects underwent a history and general physical examination, had their blood pressure checked, and were given medications depending on the health issues they discussed with the healthcare providers. The current study focused on the BP readings collected for adults aged ≥ 18 years.

People coming for general examinations stayed in a waiting area in front of the clinic to be triaged by a nurse before being checked by a physician. BPs were measured using a sphygmomanometer. Patients whose BP fell in the hypertensive range (SBP ≥ 140 mmHg, or DBP ≥ 90 mmHg) had their BP measured again once or twice by the physician, depending on the initial BP. If more than one BP was recorded, an average value was determined.

In the Gambia and Sierra Leone, one of the additional procedures performed was echocardiography using a hand-carried ultrasound (HCU) to assess left ventricular hypertrophy (LVH) to prioritise HTN treatment.^27^ LVH was previously found in 65% of people with HTN.^27^

## Statistical analysis

All the data collected during these visits, including BP measurements, medications prescribed, and diagnostic tests, were recorded on a paper form and were later entered in a computerised data spreadsheet and then de-identified. The study was reviewed and certified by the institutional review board (IRB).

Data were analysed statistically using the χ^2^-test, and the *p*-values calculated were classified based on *p* < 0.05 as considered of statistical significance. Other statistical tests included the Fisher’s exact test, Cochran–Armitage trend test, Wilcoxon rank sum test, Student’s *t*-test and ANCOVA multivariable-model test. The data were analysed by country prior to and following the combination of both data sets.

Data from Sierra Leone were available for the years 2001–2003 and 2009. Differences in SBP and DBP means were assessed across the years by analysis of covariance (ANCOVA) models. The preliminary model was a two-way full factorial model with factors gender and year and the gender-by-year interaction, and age was the covariate

In the SBP model, the gender-by-year interaction term was significant (*p* = 0.011), so separate one-way ANCOVA models were assessed in females and males, with age as the covariate. In the DBP model, the gender-by-year interaction term was not significant (*p* = 0.17); however, for comparison, separate one-way ANCOVA models were assessed in females and males, with age as the covariate. The least-squares means (LSmeans) for SBP and DBP were used to present the findings.

The data were divided into three categories: all adults with and without known recorded age (*n* = 2 615), only adults with known age ≥ 18 years old (*n* = 2 348) and only adults with known age ≥ 20 years old (*n* = 2 247). There was one female who did not have a recorded DBP.

The first classification was used to have general demographics for the whole population tested. The second and third classifications were used to observe trends of SBP, DBP and HTN prevalence with age decade, starting with 20-year-old patients. For all results including age decade analyses, the indications ≥ 70s and +70s stand for the age decade 70 years and above, which were combined together with patients over 80 years due to the small sample size in these older groups.

## Results

In total, there were 2 615 adult participants: 46.5% males (*n* = 1 215) and 53.5% females (*n* = 1 400). Because one female lacked a recorded DBP, the total number of individuals analysed based on SBP, DBP and HTN prevalence were 2 615, 2 614 and 2 614 individuals, respectively.

Of the overall population studied, 44.8% were hypertensive, while mean SBP was 133.6 ± 29.2 mmHg and mean DBP was 83.7 ± 17.0 mmHg. For females, mean SBP was 134.3 ± 29.7 mmHg and mean DBP was 84.5 ± 17.5 mmHg, while 46.2% were hypertensive. For males, mean SBP was 132.8 ± 28.5 mmHg and mean DBP was 82.8 ± 16.2 mmHg, while 43.2% were hypertensive.

The *t*-test showed no significant difference in mean SBP between males and females (*p* = 0.18). However, for mean DBP, the *t*-test indicated a significant difference between males and females (*p* = 0.008), with females having a higher mean DBP. Regarding HTN prevalence, the χ^2^-test showed that there was no significant difference between males and females, and the Fisher’s exact test confirmed this insignificance (*p* = 0.119 and *p* = 0.124, respectively).

From the total number of subjects in the study (*n* = 2 615), a large proportion (*n* = 2 348) represented individuals with known age ≥ 18 years old. The demographics of this subpopulation (Table 1) were compared across gender in terms of age, SBP and DBP means using the t-test.

**Table 1:** Characteristics of patients with known age ≥ 18 years

*Variable*	*Overall (n = 2 347)*	F (n = 1 236**)	*M (n = 1 111)*	*p-value unadjusted*	*p-value adjusted*
Age (years)	39.6 ± 16.1	38.9 ± 15.9	40.5 ± 16.4	0.018*	
SBP (mmHg)	133.2 ± 28.5	133.5 ± 28.6	132.8 ± 28.5	0.57*	0.042*
DBP (mmHg)	83.3 ± 16.7	84.0 ± 17.1	82.6 ± 16.1	0.049*	0.001*
HTN (%)	44.5	45.6	43.3	0.26†	0.016††

Values: mean ± SD or %. **Females: *n* = 1 237 for SBP and age, and *n* = 1 236 for DBP and HTN. *p*-values for M vs F: *Student’s *t*-test, ^†^χ^2^-test, ^††^multivariable model (odds ratio = 1.25). Adjustment: for age. F = females, M = males, SD = standard deviation, SBP = systolic blood pressure, DBP = diastolic blood pressure, HTN = hypertension.

For mean age, males were older on average (*p* = 0.018). For mean SBP, there was no evidence that SBP differed across gender; 133.5 mmHg for females and 132.8 mmHg for males (*p* = 0.57). However, after age adjustment, females seemed to have a significantly higher SBP compared to males; 134.1 mmHg for females and 132.1 mmHg for males (*p* = 0.042).

In the case of mean DBP, there was a small difference across gender; 84.0 mmHg for females and 82.6 mmHg for males (*p* = 0.049). After age adjustment, there was a more significant evidence of the difference in DBP; 84.3 mmHg for females and 82.2 mmHg for males (*p* = 0.001).

For HTN, the χ^2^-test showed no difference across gender (*p* = 0.26). However, after age adjustment using the multivariable model, it seemed that females had higher odds and hence risk of HTN than males (odds ratio = 1.25, *p* = 0.016).

## SBP, DBP and HTN trends

From the total number of subjects with known age in the study (*n* = 2 348), a subdivision of this population (*n* = 2 247) represented individuals with known age ≥ 20 years old. This subpopulation was used to examine the SBP, DBP and HTN prevalence trends with age decade (Table 2).

**Table 2:** Characteristics of patients with known age ≥ 20 years

*Age decade (years)*	*N*	*Gender*	*n*	*SBP ± SD (mmHg)*	*DBP ± SD (mmHg)*	*HTN % (n/N)*	*HTN Overall % (n/N)*
20s	694	F	386	119.3 ± 19.1	76.0 ± 14.0	21.8 (84/386)	22.3
		M	308	118.6 ± 16.9	74.9 ± 11.7	23.1 (71/308)^†^	(155/694)
30s	531	F	284**	125.4 ± 23.1	80.2 ± 15.4	33.8 (96/284)	33.9
		M	247	124.7 ± 21.0	79.2 ± 13.5	34.0 (84/247)^†^	(180/531)
40s	373	F	190	143.0 ± 27.6	92.6 ± 16.8	66.3 (126/190)	58.4
		M	183	136.3 ± 29.1	85.9 ± 16.8	50.3 (92/183)*	(218/373)
50s	312	F	159	151.3 ± 30.5	92.7 ± 16.3	71.7 (114/159)	69.9
		M	153	150.1 ± 32.1	91.2 ± 15.3	68.0 (104/153)^†^	(218/312)
60s	201	F	96	160.8 ± 25.5	95.7 ± 13.1	86.5 (83/96)	80.1
		M	105	155.9 ± 30.8	92.6 ± 16.8	74.3 (78/105)*	(161/201)
≥ 70s	135	F	65	158.0 ± 30.5	93.4 ± 17.9	81.5 (53/65)	75.6
		M	70	153.3 ± 27.5	91.7 ± 14.8	70.0 (49/70)^†^	(102/135)

Values: mean ± SD or % (n/N). **Females: *n* = 285 for SBP and age, and *n* = 284 for DBP and HTN. Fisher’s exact test: *significant differences, and ^†^insignificant differences. F = females, M = males, SD = standard deviation, SBP = systolic blood pressure, DBP = diastolic blood pressure, HTN = hypertension.

**Fig. 1.**
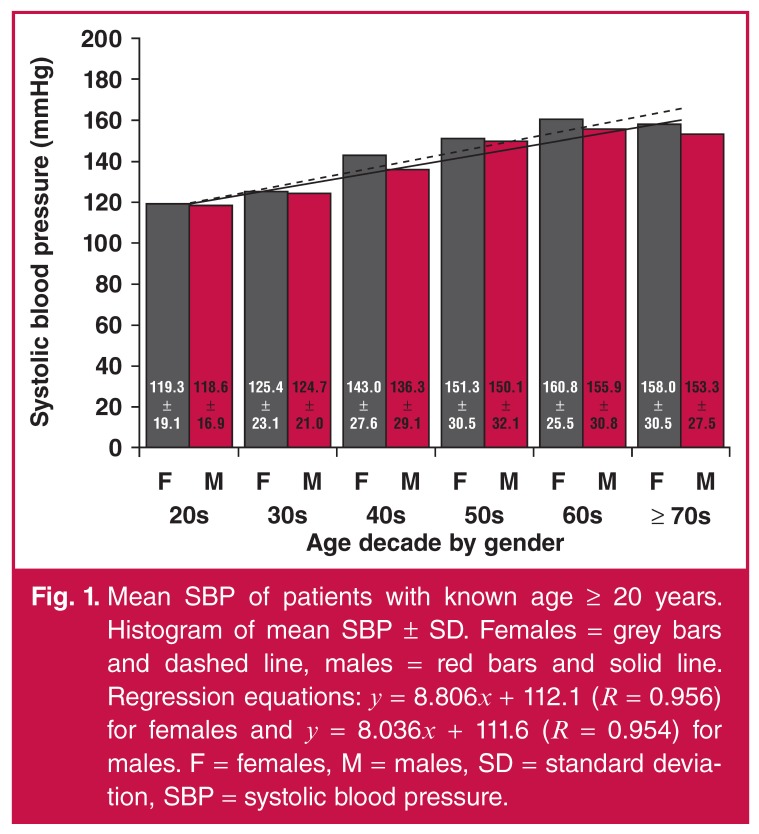
Mean SBP of patients with known age ≥ 20 years. Histogram of mean SBP ± SD. Females = grey bars and dashed line, males = red bars and solid line. Regression equations: *y* = 8.806*x* + 112.1 (*R* = 0.956) for females and *y* = 8.036*x* + 111.6 (*R* = 0.954) for males. F = females, M = males, SD = standard deviation, SBP = systolic blood pressure.

**Fig. 2.**
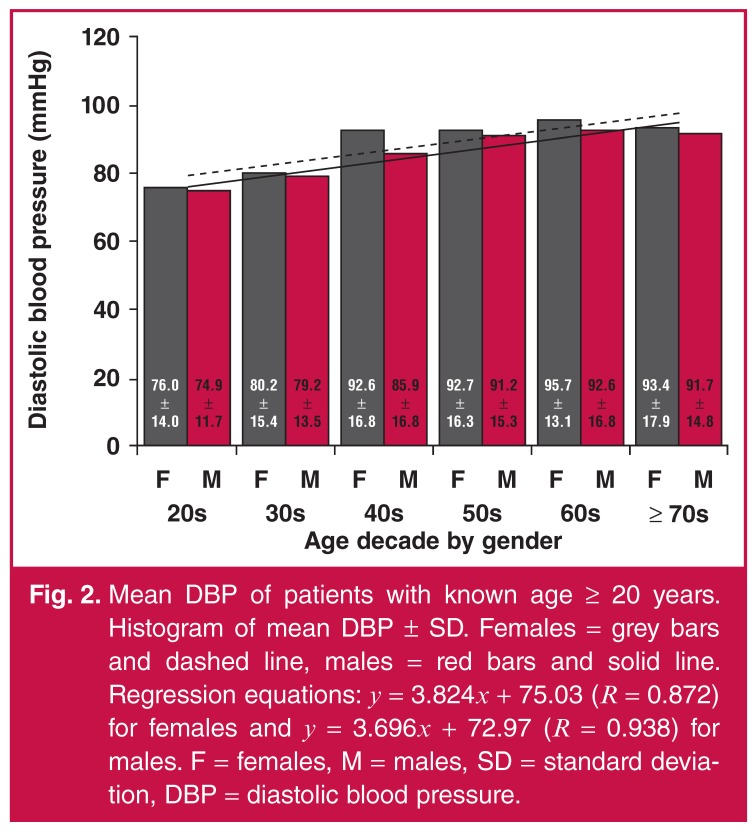
Mean DBP of patients with known age ≥ 20 years. Histogram of mean DBP ± SD. Females = grey bars and dashed line, males = red bars and solid line. Regression equations: *y* = 3.824*x* + 75.03 (*R* = 0.872) for females and *y* = 3.696*x* + 72.97 (*R* = 0.938) for males. F = females, M = males, SD = standard deviation, DBP = diastolic blood pressure.

Mean SBP increased continually with age decade for males and females (Fig. 1). The rate of increase was similar between the genders; the slopes of the regression lines for males and females were 8.036 and 8.806, respectively. As in the case of SBP, mean DBP increased continually with age decade for males and females (Fig. 2). The rate of increase was very similar between the genders; the slopes of the regression lines for males and females were 3.696 and 3.824, respectively.

**Fig. 3.**
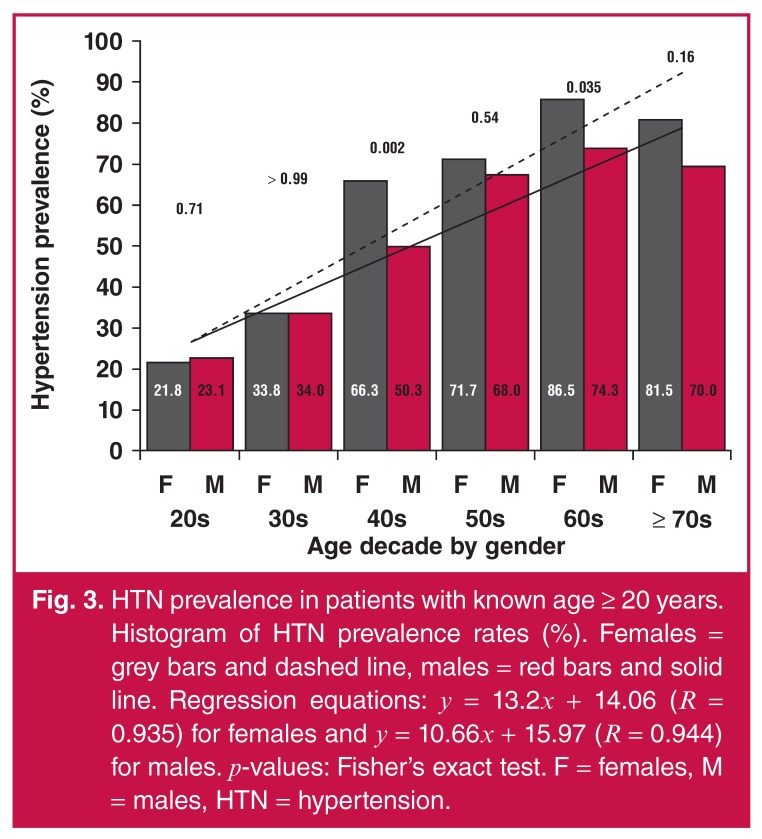
HTN prevalence in patients with known age ≥ 20 years. Histogram of HTN prevalence rates (%). Females = grey bars and dashed line, males = red bars and solid line. Regression equations: *y* = 13.2*x* + 14.06 (*R* = 0.935) for females and *y* = 10.66*x* + 15.97 (*R* = 0.944) for males. *p*-values: Fisher’s exact test. F = females, M = males, HTN = hypertension.

The Cochran–Armitage trend test showed significant differences in the HTN prevalence between each age decade, overall and gender-wise (*p* < 0.0001). This meant that within males, females, or overall scores, there was evidence that HTN prevalence increased with age decade (Fig. 3). Meanwhile, female HTN prevalence appeared to be higher than that of males in the age decades 40s, 50s, 60s, and +70s; however, the Fisher’s exact test showed evidence for the difference only in the age decades 40s and 60s (*p* = 0.002 and 0.035, respectively). The lack of significance in the +70s group could have been due to the small sample size of this age decade.

Of note, the rate of increase in HTN prevalence was somewhat different between the genders; the slopes of the regression lines for males and females were 10.66 and 13.2, respectively. In addition, there was a dramatic increase in HTN prevalence in females between the age decades 30s and 40s, compared to that in males.

## The Gambia and Sierra Leone patients

To check whether there were large differences in the demographics of subjects between the Gambia and Sierra Leone, the collected records for the year 2000 in the Gambia and the year 2001 in Sierra Leone were compared for the criteria SBP, DBP and HTN prevalence. Only the year 2001 was chosen to represent the data collected from Sierra Leone because the population sizes in the years 2000 and 2001 were comparable (Table 3).

**Table 3:** Characteristics of patients with known age ≥ 18 years in the Gambia (2000) and Sierra Leone (2001)

*Variable*	The Gambia (n = 560*)	*Sierra Leone (n = 659)*	*p-value^†^*
Age (years)	36.0 ± 15.3	39.5 ± 16.0	0.0001
SBP (mmHg)	126.7 ± 26.1	132.1 ± 24.6	0.0002
DBP (mmHg)	80.4 ± 15.8	81.5 ± 14.5	0.21

Values: mean ± SD. *The Gambia: *n* = 561 for age and SBP and *n* = 560 for DBP. ^†^Student’s *t*-test. SD = standard deviation, SBP = systolic blood pressure, DBP = diastolic blood pressure.

The χ^2^-test indicated more females and fewer males in the Gambia (*p* < 0.0001). The *t*-test showed that DBP means seemed to be similar between subjects from both countries (*p* = 0.21), while age and SBP means seemed to be different (*p* = 0.0001 and *p* = 0.0002, respectively), with Sierra Leone having higher means.

**Fig. 4.**
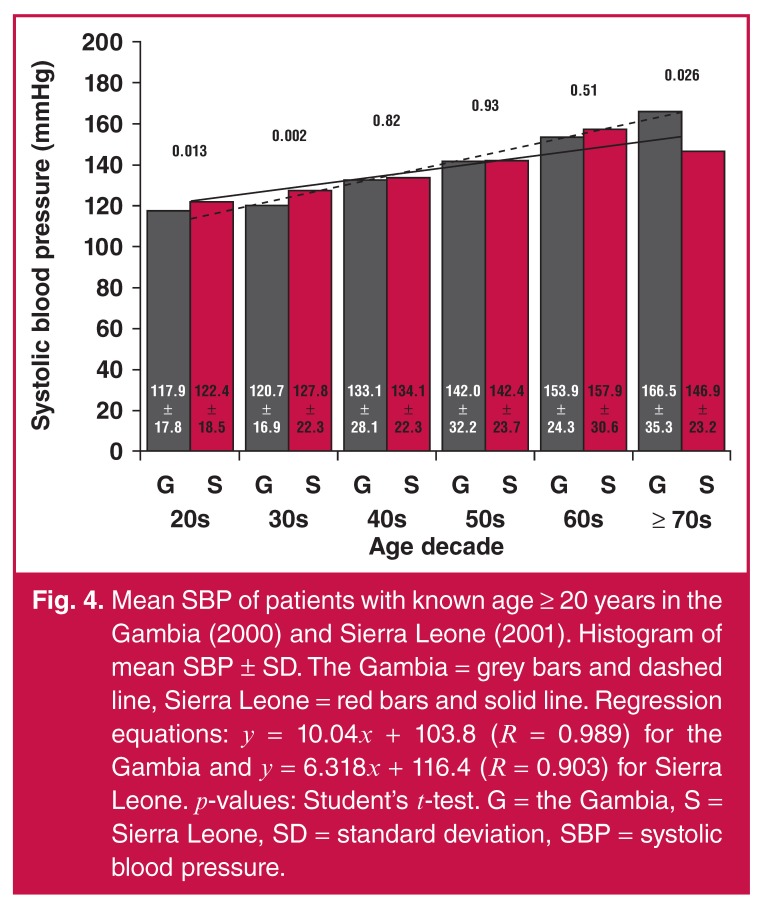
Mean SBP of patients with known age ≥ 20 years in the Gambia (2000) and Sierra Leone (2001). Histogram of mean SBP ± SD. The Gambia = grey bars and dashed line, Sierra Leone = red bars and solid line. Regression equations: *y* = 10.04*x* + 103.8 (*R* = 0.989) for the Gambia and *y* = 6.318*x* + 116.4 (*R* = 0.903) for Sierra Leone. *p*-values: Student’s *t*-test. G = the Gambia, S = Sierra Leone, SD = standard deviation, SBP = systolic blood pressure.

**Fig. 5.**
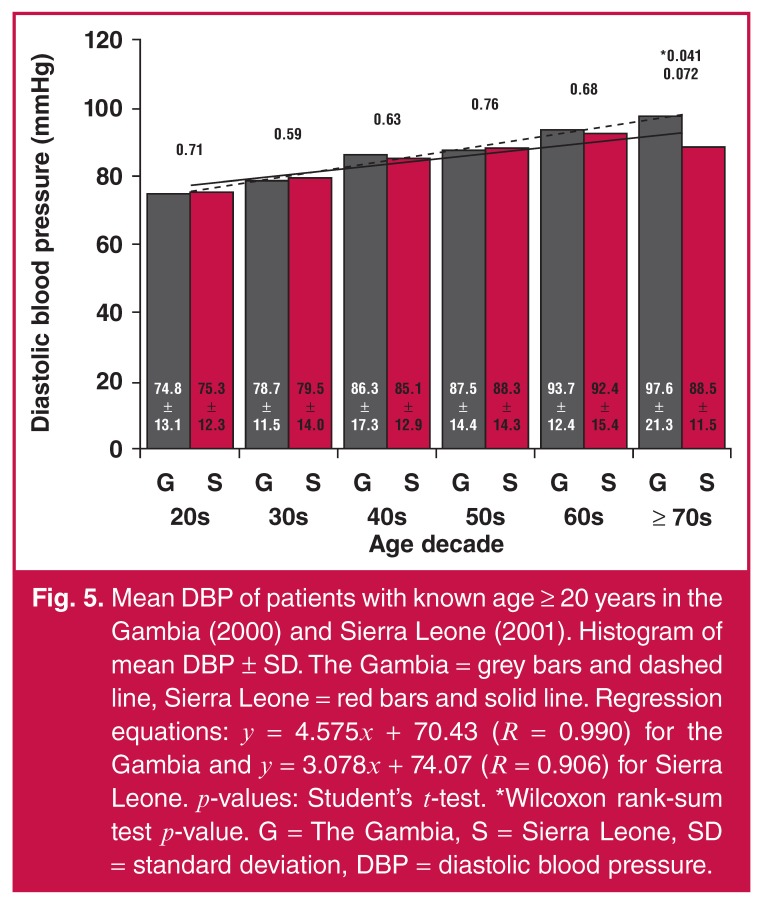
Mean DBP of patients with known age ≥ 20 years in the Gambia (2000) and Sierra Leone (2001). Histogram of mean DBP ± SD. The Gambia = grey bars and dashed line, Sierra Leone = red bars and solid line. Regression equations: *y* = 4.575*x* + 70.43 (*R* = 0.990) for the Gambia and *y* = 3.078*x* + 74.07 (*R* = 0.906) for Sierra Leone. *p*-values: Student’s t-test. *Wilcoxon rank-sum test *p*-value. G = The Gambia, S = Sierra Leone, SD = standard deviation, DBP = diastolic blood pressure.

Furthermore, SBP and DBP means continually increased with age decade for both the Gambia and Sierra Leone subjects (Figs 4 and 5, respectively). In Sierra Leone, there were higher SBP means in the age decades 20s and 30s (*p* = 0.013 and *p* = 0.002, respectively) and lower SBP means in the age decade ≥ 70s (*p* = 0.026) in comparison with SBP means in the Gambia, as shown in Fig. 4.

The increase in mean SBP seemed to be faster in the Gambia when compared with Sierra Leone, based on the regression line slopes of 10.04 and 6.32, respectively (Fig. 4). Similarly, the increase in mean DBP seemed to be faster in the Gambia when compared with Sierra Leone, based on the regression line slopes of 4.58 and 3.08, respectively (Fig. 5). As shown in Fig. 5, DBP mean in the Gambia was higher than in Sierra Leone in the age decade ≥ 70s (*p* = 0.041). The Wilcoxon test was more trusted for the small sample size, which was the case in the age decade ≥ 70s.

**Fig. 6.**
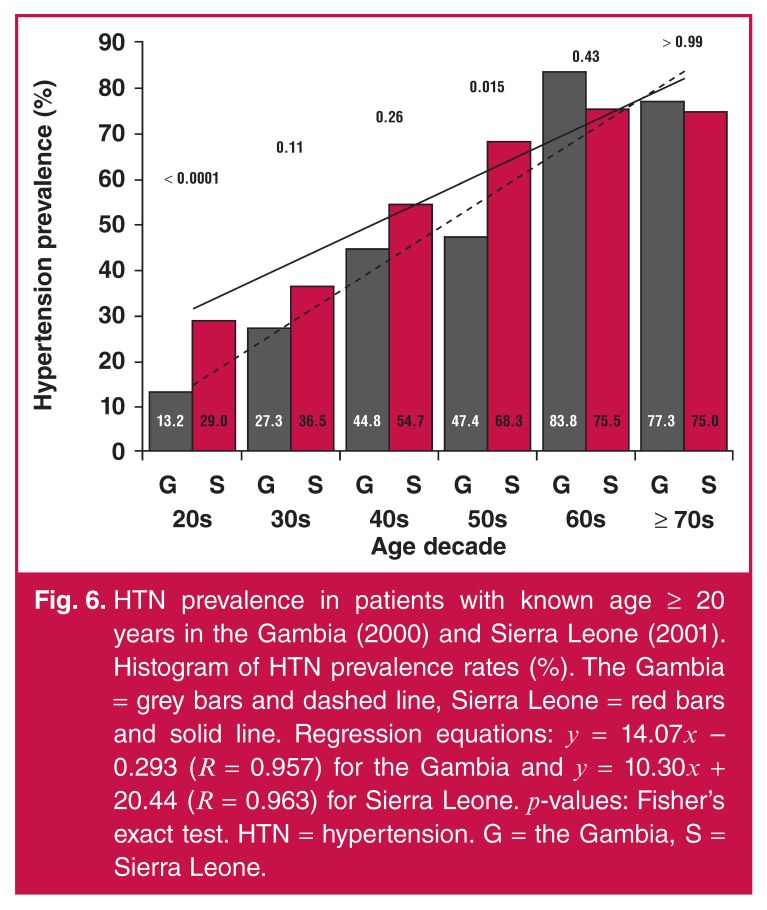
HTN prevalence in patients with known age ≥ 20 years in the Gambia (2000) and Sierra Leone (2001). Histogram of HTN prevalence rates (%). The Gambia = grey bars and dashed line, Sierra Leone = red bars and solid line. Regression equations: *y* = 14.07*x* – 0.293 (*R* = 0.957) for the Gambia and *y* = 10.30*x* + 20.44 (*R* = 0.963) for Sierra Leone. *p*-values: Fisher’s exact test. HTN = hypertension. G = the Gambia, S = Sierra Leone.

HTN prevalence appeared to be continually increasing with age decade for both the Gambia and Sierra Leone (Fig. 6). However, this increase seemed to be occurring at a faster rate in the Gambia than in Sierra Leone, as detected by the trend line slopes of 14.07 and 10.30, respectively. In addition, HTN prevalence in Sierra Leone was higher in the age decades 20s and 50s (*p* < 0.0001 and *p* = 0.015, respectively) compared to HTN prevalence in the Gambia.

Overall, among adults with known age ≥ 20 years old, the HTN prevalence rates in the Gambia in 2000 and in Sierra Leone in 2001 were 32.4 and 46.6%, respectively, while the Fisher’s exact test showed a significant difference between both values (*p* < 0.0001). The Cochran–Armitage trend test showed a significant difference between the HTN prevalence of each age decade by country (*p* < 0.0001).

## Sierra Leone patients

To check whether there was a trend in the data collected in Sierra Leone over the years 2001–2003 and 2009, ANOVA was performed on SBP and DBP LSmeans, adjusted for the relationship with age and separated by gender (Table 4). Adjusted for age, SBP LSmean in females was similar between 2009 and 2003 (*p* = 0.84), higher in 2003 than in 2001 (*p* = 0.003), and higher in 2001 than in 2002 (*p* = 0.014). DBP LSmean in females was higher in 2003 than in 2009 (*p* = 0.0002), similar between 2009 and 2001 (*p* = 0.13), similar between 2001 and 2002 (*p* = 0.35), and lower in 2002 than in 2009 and 2003 (*p* = 0.029 and *p* < 0.0001, respectively).

**Table 4:** Characteristics of patients with known age ≥ 18 years in Sierra Leone

*Data collection year*	*Gender*	*N*	*SBP (mmHg)*	*p-values*	*DBP (mmHg)*	*p-values*
2001*	F	297	135.1	0.014, 0.003	83.0	0.35, < 0.0001
	M	362	131.7	0.73, < 0.0001	81.3	0.41, < 0.0001
2002**	F	304	130.1	< 0.0001, < 0.0001	81.8	< 0.0001, 0.029
	M	359	132.3	< 0.0001, 0.002	82.2	< 0.0001, 0.068
2003^†^	F	209	141.8	0.84	92.6	0.0002
	M	74	150.7	0.043	95.9	< 0.0001
2009^††^	F	108	142.4	0.010	85.6	0.13
	M	74	142.4	0.0009	85.5	0.022

Values: least squares means (LSmeans). *p*-values: *2001 vs 2002 and 2003, respectively, **2002 vs 2003 and 2009, respectively, ^†^2003 vs 2009, ^††^2009 vs 2001. F = females, M = males, SBP = systolic blood pressure, DBP = diastolic blood pressure.

After age adjustment, SBP LSmean in males was higher in 2003 than in 2009 (*p* = 0.043), higher in 2009 than in 2002 (*p* = 0.002), and similar between 2002 and 2001 (*p* = 0.73). DBP LSmean in males was higher in 2003 than in 2009 (*p* < 0.0001), similar between 2009 and 2002 (*p* = 0.068), similar between 2002 and 2001 (*p* = 0.41), lower in 2001 than in 2009 and 2003 (*p* = 0.022 and *p* < 0.0001, respectively). To summarise, SBP and DBP LSmeans were generally higher in 2003 and 2009 compared to those in 2001 and 2002.

## Discussion

## SBP, DBP and HTN trends

Mean SBP was shown to increase with age decade in both males and females (Fig. 1). There was a significant difference in mean SBP between the genders after age adjustment, with females having a higher mean SBP. Previous studies in Kenya, Tanzania, the Gambia and West Africa showed an increase in SBP with increasing age in both genders.^1^,^21^,^23^,^28^ The study in Tanzania showed that the increase in mean SBP with age was steeper in females.^21^

Mean DBP increased with age decade and then plateaued as age decade reached +70s in both males and females (Fig. 2), which was similar to a previous study in the Gambia.^23^ Prior studies showed that mean DBP increased with age and then plateaued by ages 45–54 and 55–64 years in Tanzania and West Africa, respectively.^21^,^28^ Our study showed that females had a higher mean DBP than males after age adjustment.

HTN prevalence was shown to increase with age decade for both males and females (Fig. 3). Previous studies in Sierra Leone, Kenya and West Africa showed that HTN prevalence rates increased with age in both genders.^1^,^28^,^29^ Comparing males to females, we found that females had higher odds and risk of HTN than males. Similarly, studies in Tanzania and Uganda showed that HTN was significantly higher in females.^21^,^30^ This may have been due to post-menopausal hormonal changes.^31^ Females showed a relatively higher HTN prevalence, starting with the age decade 40s and above (Fig. 3), consistent with post-menopausal hormonal changes related to the observed increase in androgen levels post menopause.^32^

Knowing that obstructive sleep apnoea/hypopnoea syndrome (OSAHS) is a risk factor for developing HTN, post-menopausal women with OSAHS showed a higher prevalence of HTN when compared to those without OSAHS and to all pre-menopausal women.^33^ It was also noted in the same study that among females with OSAHS, post-menopausal women had higher SBP and DBP averages when compared to pre-menopausal women. This may have been due to falling oestrogen levels in post-menopausal women, because oestrogen decline causes a rise in BP via the activation of the renin–angiotensin system, which in turn explains the observed higher plasma renin levels in post-menopausal females compared to males and pre-menopausal females.^32^

Furthermore, endothelin levels are higher in post-menopausal females, which explains in part the observed higher BPs, since endothelin causes sodium re-absorption, which in turn causes higher BP.^32^ All of these factors make increasing age a risk factor of acquiring HTN in females, considering also the observation that about 60% of females aged > 65 years are hypertensive.^32^

## HTN in Sierra Leone and the Gambia

This study highlights the high prevalence of HTN in the Gambia and Sierra Leone. HTN seems to be highly prevalent as a CVD in the sub-Saharan African region,^19^ and may be rising over time. In 2006, a cross-sectional study in Uganda revealed that 252 individuals out of the 842 participants (29.9%) were hypertensive.^30^ In 2007–2008, a study in Kenya found that 50.1% of 4 396 subjects were hypertensive.^1^ In 1991–1995, HTN prevalence in rural and urban Cameroon was 17.3%; however, in 2003, the rate rose by an additional 7.3%.^34^

HTN in Sierra Leone was reviewed in several studies. Between 1983 and 1992, HTN accounted for about 7.5% on average of all deaths in Freetown, the capital of Sierra Leone.^35^ A retrospective study, published in 1993, showed that among 87 subjects, 59 individuals were hypertensive.^36^

HTN prevalence, according to the HTN definition of ≥ 160/95 mmHg, was measured in four Sierra Leonean towns and villages. In 1998, in Njala Komboya and Kychum, HTN prevalence was 24.8 and 17.6%, respectively.^37^ Similarly, in 1999, HTN prevalence was 23.4 and 14.7% in Freetown and Port Loko, respectively.^38^ Recently, in Bo in 2009, 25.2% of 3 944 individuals aged ≥ 15 years old were hypertensive according to the HTN definition of ≥ 140/90 mmHg; however, the study showed no difference in BPs between males and females.^29^ HTN prevalence by calendar year seems to agree with our results, showing that SBP and DBP LSmeans tended to be higher in the later years (2003 and 2009) than in the earlier years (2001 and 2002).

Several studies reviewed HTN in the Gambia. In 1996–1997, the HTN prevalence, according to the definition of ≥ 160/95 mmHg, was 7.1%, whereas by 1998, it rose to 10.2%, an increase of 3.1% in a year.^34^ According to the HTN definition in the current study (≥ 140/90 mmHg), van der Sande showed in 1997 that 24.2% of 6 048 individuals in the Gambia were hypertensive.^23^ Although the prevalence of HTN seems to be high in the Gambia, a study in 2001 pointed out that HTN prevalence in the Gambia varies with the specific geographical area in the country.^39^

These results show the high prevalence rate of HTN in the Gambia and Sierra Leone. Comparatively, in our current study, the HTN prevalence rate in both countries combined was 46.2% among females (*n* = 1 399), 43.2% among males (*n* = 1 215), and 44.8% overall (*n* = 2 614).

## Influence of low SES

One major dilemma in sub-Saharan Africa is the low SES of countries in the region, including the Gambia and Sierra Leone. It was estimated that the total number of hypertensive adults in developing countries in 2000 was 639 million, compared to 333 million in developed countries,^20^ which is a result of the difference in SES.^18^

The low SES establishes a variety of factors contributing to the prevalence of HTN, including a low HTN treatment rate, low levels of education and awareness, high salt and low potassium intakes, as well as an increased stress level. All these factors contribute directly or indirectly to the HTN prevalence rate among countries in the sub-Saharan African region.^18^,^34^ Evidently, low SES was linked to high BP means, with a stronger effect on females than males.^40^

## HTN treatment and SES

The treatment rate of a chronic disease depends on several factors, including the cost of the treatment associated with the disease. As mentioned, the SES of Sierra Leone and the Gambia is low and this may contribute to lack of availability of antihypertensive treatment.^18^ A study in Kenya showed that only 15% of hypertensive individuals were able to obtain treatment for HTN.^1^ A low SES contributed to the government not having adequate amounts of medications to distribute among patients.

In a 1999 survey in Cape Town, South Africa, 15.5% of patients reported that during filling prescriptions, insufficient medication was supplied.^41^ A low SES also contributed to individual patients not having enough income to pay for the medications. In the Gambia, in 2006, the rate of unemployment was high.^42^ Therefore, the inability to obtain medication was a factor contributing to the high HTN prevalence rate.

## Education levels and SES

The awareness of HTN was previously correlated with the prevalence rate of the disease.^22^ This awareness is usually provided by schools as well as public healthcare facilities. Establishment of schools has been difficult in societies with low SES. Concerning school education, in the Gambia, a research study showed that 10 and 56% of women aged 10–25 (*n* = 50) and 35–50 (*n* = 50) years, respectively, were unable to read, whereas 34% of 50 males aged 35–50 years were unable to read.^42^ A study in Tanzania also pointed out that SBP was associated with education, which in turn was associated with SES; the higher the SES, the lower the SBP.^21^

Establishment and funding of public healthcare facilities, such as medical schools and nursing schools, has also been difficult in low SES countries. In 2000–2010, there were 0.4 physicians and 5.7 nurses and midwives per 10 000 individuals in the Gambia, while in Sierra Leone, there were 0.2 physicians and 1.7 nurses and midwives per 10 000 individuals. On the other hand, in the USA, there were 26.7 physicians and 98.2 nurses and midwives per 10 000 individuals.^26^

These low healthcare provider-to-population ratios (61/100 000 in the Gambia and 19/100 000 in Sierra Leone) reflect the inadequate establishment and funding of public healthcare facilities in these countries. It is estimated that by 2015, according to the needs-based model, there will be a total of 45 countries in the world with physician shortage, 32 countries (~ 70%) of which are in Africa.^43^

## Potassium and sodium levels and SES

HTN is related to sodium and potassium levels based on renin secretion, cellular sodium–potassium pumps and therefore the individual’s nephron mass. The effect of sodium and potassium levels on HTN in the Gambia and Sierra Leone depends on two factors: the intrinsic propensities of the individual being of African descent and the individual’s levels of salt intake, as well as vegetable and fruit (potassium) intake. Research in the USA and Europe illustrated that people of African descent had higher HTN prevalence and were at a higher risk of acquiring organ damage due to HTN,^23^ in part because of lower nephron mass, macula densa mass, sodium detection levels and sodium–potassium pump activity.^17^,^44^,^45^

It was found that higher SBP and DBP means were apparent in individuals with higher sodium intake levels when compared with either intermediate or low sodium intake.^46^ It has been shown that there is a linearly increasing correlation between sodium intake and HTN prevalence and mean SBP.^47^,^48^ Globally, it was estimated that sodium intake in children older than five years of age was in excess by about 100 mmol/day.^49^ This was a significantly high sodium intake level, considering that a high level of salt intake in infancy and childhood correlated to a high BP later in life.^50^,^51^ In central and South Africa, it was found that sodium levels in cells and in circulating blood were high in hypertensive individuals.^18^

In the Gambia, intake of salt-preserved foods was high due to inadequate refrigeration. As a consequence, there was a high salt and sodium intake.^23^ A low SES reduces the likelihood for a household to own a refrigerator and to receive electricity. In the Gambia, 14% of 50 females aged 14–25 years and 34% of 50 males aged 35–50 years did not receive electricity at home.^42^ This electricity grid showed inadequate electricity reception in Gambian households, leading to an inability to refrigerate foods.

Furthermore, dietary potassium intake was related to BP.^52^ Studies compared sodium to potassium intake and showed that in lower SES communities, the ratio between sodium and potassium intakes was high; however, the situation was nearly reversed in higher SES groups because potassium intake was higher than that in lower SES groups.^40^ In Ghana, it was shown that an insufficient fruit and vegetable (a source of potassium) intake in 39.6% of males and 38.2% of females was considered a factor contributing to HTN prevalence.^53^ Therefore, this low potassium intake assists in maintaining a high HTN prevalence rate.

## Psychosocial status and SES

One of the factors contributing directly to an increased HTN prevalence is the psychological status of the individual, affected by stressors correlating with low SES. Studies have shown that stress, economic transition, and high BP may be correlated.^25^,^54^

As noted by a recent study, there is a significant association between psychosocial stress and endothelial dysfunction, which contributes to the development of CVD.^24^ The study found that cold stress caused a more prominent increase in DBP in white South Africans compared to black South Africans. In addition, black Africans who reported higher levels of psychosocial distress had lower L-arginine/ADMA (asymmetric dimethylarginine) ratio. ADMA is known to be an inhibitor of the endothelial NO synthase, which produces NO from L-arginine. The study concluded that psychological distress significantly affects the L-arginine/NO system, with some ethnic differences.^24^

In a low-SES community, employment levels are very low, therefore leading to an increase in stress levels for individuals in a household due to the lack of an income source to support a living. Another possible source of stress in the Gambia and Sierra Leone was the instability in both societies. The instability in Sierra Leone was due to the persisting civil war from 1991 to 2002, while in the Gambia, it was due to their increasing potential population due to the outmigration of Sierra Leoneans to surrounding countries, one of which was the Gambia. This instability could have served as a psychological stressor that led to the increase in HTN prevalence in both populations.

## Additional HTN risk factors

Additional possible risk factors for HTN include smoking, alcohol consumption, schistosomiasis specifically in the Gambia, and certain genotypic correlations. Smoking is associated with CVD and increases HTN risk by two- to three-fold.^55^ In the Gambia, by 2006, 29.2% of males and 2.6% of females were tobacco smokers, compared to the USA, where 25.4% of males and 19.3% of females were tobacco smokers.^26^

Alcohol intake also contributes to a higher BP and the prevalence of HTN.^56^ A study in Uganda considered past and present alcohol intake as a risk factor associated with HTN prevalence.^30^ As for the Gambia and Sierra Leone, by 2005, the total alcohol consumption among adults aged ≥ 15 years in the Gambia was 2.4%, while in Sierra Leone, it was 6.5%, compared to 8.5% in the USA.^26^

In the Gambia, schistosomiasis was another risk factor. Studies showed that prevalence of diastolic hypertension in adults was two- to four-times higher in *Schistosoma haematobium* endemic areas,^23^ including parts of the Gambia and Sierra Leone.

Regarding genotypic influences, recent studies relate certain loci and some single nucleotide polymorphisms in the human genome to BP and HTN. An admixture mapping study identified a probable relatioship between chromosomes 6q24 and 21q21 and HTN risk in African Americans.^57^ These two regions included two loci on chromosome 21q, and five other markers on chromosome 6q that suggested a genetic linkage to elevated BP.

One genome-wide association study (GWAS) related three genes, previously associated with BP in Americans of European descent, with BP in African Americans.^58^ The three genes were *SH2B3, TBX3-TBX5, and CSK-ULK3*, all of which are genetic variants influencing BP in African Americans, and generally in people of Africa descent.

## Influences of lifestyle and economic development

**Fig. 7.**
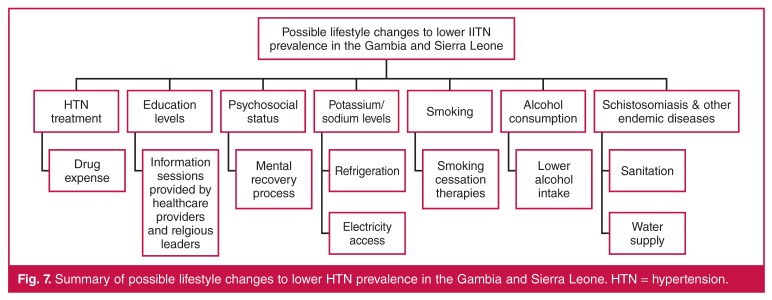
Summary of possible lifestyle changes to lower HTN prevalence in the Gambia and Sierra Leone. HTN = hypertension.

Lifestyle changes, including smoking cessation, lower alcohol intake, sanitation, clean water supply, refrigeration, and electricity access may influence HTN prevalence (Fig. 7). The ability to make lifestyle changes may be related to SES, educational level and economic development.

The economic development of both countries could raise both countries’ SES, thus diminishing several factors contributing to HTN prevalence. Sources of economic development in the Gambia and Sierra Leone could include the many natural resources present in these countries, which could be used for economic self-sufficiency. With economic development, both governments could offer funds for healthcare systems to lower HTN prevalence.

Concerning HTN awareness, healthcare providers in healthcare centres and religious leaders in religious institutions could routinely make people aware of the disease, its progression and burden, and its preventive means.^53^ The government could also establish national policies and programmes so that all individuals, whether educated or not, would have an idea about the existence of the disease HTN. In the Gambia, an improvement in education and disease awareness is already underway.^42^

Increased potassium (vegetable/fruit) intake and lowered sodium intake are needed for protection against HTN.^18^,^20^,^46^ The high sodium (salt) intake is mainly due to the unavailability of food preservation via refrigeration. Affordable electricity systems could be established using the Berra Kunda waterfalls in the Gambia on the border with Senegal, and the Bumbuna waterfalls in Sierra Leone for hydroelectric power.

Chronic financial stress related to low SES and poor economic conditions is potentially modifiable. A study in Ghana and Cameroon suggested that religious institutions and leaders should encourage the people to overcome their financial problems and to start a recovery process from their stress.^53^

Considering schistosomiasis, the Gambia and part of Sierra Leone fall within the endemic region of the disease.^23^,^59^ The main prevention against such parasitic diseases is the improvement of drinking water sources and sanitation facilities. Improper sanitation and water supply are related to ascariasis, diarrhoea, trachoma, schistosomiasis and other diseases.^60^ An analysis showed that cleaner water supplies led to a median reduction in schistosomiasis morbidity rate of 69% for all studies and 77% for four selected rigorous studies.^60^

## Study limitations

Study limitations include the gap in data collection in Sierra Leone since the data were collected from 2001 to 2003 and in 2009. The data from the Gambia were only collected in 2000, which may result in a smaller sample size from the Gambia contributing to the findings of the study. In addition, combining the data collected from both countries could potentially be a weakness in the study, taking into account the fact that there were some minor differences between the data collected from the Gambia in 2000 and from Sierra Leone in 2001, as discussed above. Finally, some ages were missing from the records, resulting in the exclusion of these individuals from the statistical analyses involving age.

## Conclusion

HTN was highly prevalent in the Gambia and Sierra Leone. This may have been due to low HTN treatment rates, low education and awareness levels, low potassium and high sodium intakes, and high stress levels, all of which are part of the persistently low SES in both countries. Additional risk factors include smoking, alcohol consumption, identified genetic loci and endemic diseases. Lifestyle changes need to be instituted to lower this high prevalence of HTN. Changes include raising the awareness of the disease, initiating a stress-recovery process, finding alternative ways to preserve foods and improving sanitation and water supply sources.
